# Measurement of Lipid Accumulation in *Chlorella vulgaris* via Flow Cytometry and Liquid-State ¹H NMR Spectroscopy for Development of an NMR-Traceable Flow Cytometry Protocol

**DOI:** 10.1371/journal.pone.0134846

**Published:** 2015-08-12

**Authors:** Michael S. Bono Jr., Ravi D. Garcia, Dylan V. Sri-Jayantha, Beth A. Ahner, Brian J. Kirby

**Affiliations:** 1 Sibley School of Mechanical and Aerospace Engineering, Cornell University, Ithaca, NY 14853, United States of America; 2 Biological and Environmental Engineering, Cornell University, Ithaca, NY 14853, United States of America; 3 Division of Hematology and Medical Oncology, Weill Cornell Medical College, New York, NY 10065, United States of America; Royal Netherlands Institute of Sea Research (NIOZ), NETHERLANDS

## Abstract

In this study, we cultured *Chlorella vulgaris* cells with a range of lipid contents, induced via nitrogen starvation, and characterized them via flow cytometry, with BODIPY 505/515 as a fluorescent lipid label, and liquid-state ^1^H NMR spectroscopy. In doing so, we demonstrate the utility of calibrating flow cytometric measurements of algal lipid content using triacylglyceride (TAG, also known as triacylglycerol or triglyceride) content per cell as measured via quantitative ^1^H NMR. Ensemble-averaged fluorescence of BODIPY-labeled cells was highly correlated with average TAG content per cell measured by bulk NMR, with a linear regression yielding a linear fit with *r* ^2^ = 0.9974. This correlation compares favorably to previous calibrations of flow cytometry protocols to lipid content measured via extraction, and calibration by NMR avoids the time and complexity that is generally required for lipid quantitation via extraction. Flow cytometry calibrated to a direct measurement of TAG content can be used to investigate the distribution of lipid contents for cells within a culture. Our flow cytometry measurements showed that *Chlorella vulgaris* cells subjected to nitrogen limitation exhibited higher mean lipid content but a wider distribution of lipid content that overlapped the relatively narrow distribution of lipid content for replete cells, suggesting that nitrogen limitation induces lipid accumulation in only a subset of cells. Calibration of flow cytometry protocols using direct *in situ* measurement of TAG content via NMR will facilitate rapid development of more precise flow cytometry protocols, enabling investigation of algal lipid accumulation for development of more productive algal biofuel feedstocks and cultivation protocols.

## Introduction

The development of rapid, accurate methods to measure the lipid content of algae cells is important to the success of biodiesel production from this promising biomass resource [1, 2]. Algae cells can accumulate high quantities of lipids, especially when subjected to environmental stresses such as nitrogen limitation. During environmental stress, neutral lipids in the form of triacylglycerides (TAGs) can accumulate up to 20–50% of dry cell weight [3] and are easily converted to biodiesel via transesterification [2]. Because TAG biosynthesis is enhanced when algae are subjected to stresses that frequently also inhibit cell growth, algal lipid content can vary widely with growth conditions and over time [1, 2]. This variability necessitates time-series measurement of lipid content for different growth conditions in order to improve cultivation protocols and monitor changes in lipid content during industrial production [1, 4]. Moreover, during the screening of algae strains, investigation of lipid synthesis for genetic modification of existing strains, and development of cultivation protocols, it is particularly beneficial to characterize algal lipid content at the single-cell level. Algae cells in culture exhibit a distribution of lipid contents for the same culture conditions [5], even for isogenic cultures [6]. Single-cell lipid measurement facilitates sorting of cells with high lipid content for the development of more productive algae strains [7] and fundamental investigation of the dynamics of algal lipid accumulation [6], yielding knowledge that will enable genetic engineering of improved strains [8, 9].

Algal lipid content can be measured using a variety of methods, including direct measurement via extraction and gravimetric determination [10, 11]; *in situ* spectroscopy via Fourier-transform infrared spectroscopy (FTIR) [12], Raman microspectroscopy [6], or nuclear magnetic resonance [4, 13–16]; electrokinetic characterization via dielectric spectroscopy [17] or dielectrophoresis [18–21]; and using fluorescence in bulk fluorometry [22] or flow cytometry [23, 24] of algae cells labeled with fluorescent lipid probes. Among these, flow cytometry is the most widely used analytical technique capable of characterizing algal lipid content with single-cell resolution. Flow cytometric instrumentation is inexpensive and widespread, and well-defined protocols exist for measuring algal lipid content simultaneously with other parameters such as cell size, biomass, internal complexity, chlorophyll autofluorescence, and enzyme activity at the single-cell level [24, 25]. In addition, flow cytometry is capable of rapid characterization (< 1 hr) and cell sorting in order to identify productive strains and prepare axenic cultures [7, 24].

Algal lipid content has been measured via flow cytometry of cells labeled with Nile Red [23, 24, 26]. The excitation and emission maxima of Nile Red shift to lower wavelengths as the polarity of the environment surrounding the dye decreases, yielding a fluorescent probe that can be used for quantitation of neutral lipids, polar lipids, or the ratio of polar to neutral lipids in algae cells [26]. However, as Nile Red does not specifically accumulate within lipid deposits, it can exhibit non-specific fluorescence when bound to proteins and other cellular components [23, 27]. In addition, Nile Red fluorescence emission can overlap with that of chlorophyll autofluorescence [24, 28], resulting in spectral interference that complicates measurement. Nile Red uptake varies widely between cells, depending on the structure of the cell wall, and the fluorophore has limited photostability [22, 28]. As a result, accurate measurement often requires monitoring the Nile Red fluorescence over time in order to measure the maximum fluorescence [17, 29, 30].

Because of these limitations, the fluorophore BODIPY 505/515 (4,4-Difluoro-1,3,5,7-Tetramethyl-4-Bora-3a,4a-Diaza-s-Indacene) has been investigated as an alternative fluorescent label for algal lipid deposits [23, 24, 28, 29]. Unlike Nile Red, BODIPY exhibits uniform excitation and emission characteristics relatively independent of the pH and polarity of its surrounding environment [23, 28]. Instead of differing in emission characteristics based on the polarity of the surrounding environment, BODIPY accumulates in hydrophobic environments because of its high oil/water partition coefficient [29], resulting in fluorescent labeling of algal lipid deposits with greater specificity than Nile Red [28]. In addition, BODIPY fluorescence emission is significantly offset from chlorophyll autofluorescence, allowing for measurement without spectral interference [24]. BODIPY has good photostability and is easily transported across cell membranes because of its high oil/water partition coefficient, resulting in fluorescence that is relatively constant after cellular uptake and removing the need for time-series monitoring of cellular fluorescence [28]. These characteristics make BODIPY a promising fluorophore for measuring single-cell algal lipid content.

As flow cytometry is an indirect measurement of lipid content via the fluorescence of lipid probes such as Nile Red or BODIPY, it must be calibrated to a direct measurement of lipid content. Currently, flow cytometry protocols are calibrated to lipid content measured via extraction [23]. However, extraction of algal lipids results in fractional losses before analysis [4], and the amount of lipid extracted varies with the method of extraction [31]. In addition, extraction of algal lipids using organic solvents may result in co-extraction of non-lipid components such as proteins and pigments, resulting in an overestimation of cellular lipid content when measured via simple gravimetric determination and necessitating additional steps such as transesterification and/or chromatographic separation in order to ensure that only lipids are quantitated [1, 32, 33]. As a result, historically algal lipid measurement via extraction has required a complicated, time-consuming process for accurate measurement [10, 11, 13], although recently developed techniques for rapid extraction have somewhat decreased the required time and complexity [34]. Because flow cytometry measures the fluorescence of lipid probes within intact algae cells, the aforementioned fractional losses and possible nonspecific extraction result in an inherent discrepancy between the quantity of lipids sampled via flow cytometry and extraction. We expect that calibration via direct *in situ* measurement of lipid deposits in intact algae cells will avoid this discrepancy, facilitating more precise calibration of flow cytometry protocols for quantification of lipid content in algae cells and other biological samples.

Nuclear magnetic resonance (NMR) spectroscopy is capable of determining the composition of complex biological mixtures with minimal sample preparation that generally avoids the complexity of chromatographic separation or chemical derivatization [35]. NMR spectroscopy can interrogate the interior of intact cells, enabling characterization of lipids and other metabolites within whole algae cells [14, 15, 36, 37]. Of particular interest to this study, ^1^H NMR spectroscopy has been used to quantitate intracellular TAG deposits within intact cells in biological tissues [38]. Recently, Davey et al. [4] quantitated TAGs in live microalgal cultures in their native growth media using liquid-state ^1^H NMR spectroscopy and the quantitative NMR method developed by Henderson [39]; in this method, the reference compound is contained in coaxial inserts in order to avoid interactions between the reference and sample solutions. These results demonstrate that ^1^H NMR spectroscopy is capable of *in situ* quantification of lipid deposits in intact cells, making it an appropriate calibration method for flow cytometry of algae cells labeled with fluorescent lipid probes. Moreover, the ease of sample preparation required for NMR spectroscopy makes it ideal for high-throughput calibration of flow cytometry protocols for different algae species. As lipid probe fluorescence varies between algae strains [28], high-throughput calibration of flow cytometry protocols for newly-developed algae strains is necessary for quantitative assessment of lipid accumulation dynamics in these new strains.

In this work, we demonstrate the utility of NMR calibration of single-cell flow cytometry measurements. We characterized algae cultures with a range of lipid contents via flow cytometric measurement of cells labeled with the fluorescent lipid probe BODIPY 505/515, and compared the resulting single-cell fluorescences with average TAG content per cell measured at the culture level via liquid-state ^1^H NMR spectroscopy. We expect that the use of NMR spectroscopy for *in situ* direct measurement of lipid content will facilitate more accurate calibration of flow cytometry protocols based on the fluorescence of lipid probes within intact algae cells. As calibrated flow cytometry protocols can measure lipid content at the single-cell level, they provide valuable information on the distribution of lipid accumulation within a cell culture that can be used to investigate mechanisms for lipid accumulation dynamics, sort productive cells for improved strain development, or identify superior cultivation protocols for maximum biofuel production.

## Materials and Methods

### Algae culture

*Chlorella vulgaris* Beijerinck (UTEX 259) was acquired from the UTEX Culture Collection of Algae at The University of Texas at Austin and maintained in liquid TAP medium cultures. Media for primary and experimental cultures were acquired and prepared as described in Bono et al. [17] based on earlier formulations for replete and nitrogen-free TAP media [40, 41]. Experimental cultures were prepared by inoculating 500 mL of TAP medium with 1.4 mL liquid primary culture and cultivating for 14 days at 24±1°C, a photon flux density of 150±20 *μ*mol/m^2^s, continuous lighting, and no stirring or shaking. These conditions were found to result in a growth rate of 0.26±0.01 days^−1^ and a biomass concentration of 0.6 g/L during stationary phase (S1 Fig). We modulated the lipid accumulation by resuspending the *Chlorella vulgaris* in fresh nitrogen-replete or nitrogen-limited TAP medium and cultivating resuspended cells in 100-mL cultures for an additional 3–4 days at the culture conditions described above, resulting in the cultures described in Table 1. All other experimental details are as described in Bono et al. [17].

**Table 1:** Culture conditions and measurements for each culture.

Days after resuspension	Culture medium	Mean BODIPY fluorescence (FIU)	Cell conc. (10^6^ cells/mL)	NMR TAGs (*μ*g/mL culture)	NMR TAGs (fg/cell)
3	Replete	2500 ± 300	104 ± 6	20 ± 4	190 ± 40
3	N-limited	4300 ± 400	70 ± 4	51 ± 2	740 ± 50
4	Replete	1900 ± 200	137 ± 7	13 ± 2	100 ± 20
4	N-limited	5700 ± 600	56 ± 3	62 ± 4	1100 ± 90

### Flow cytometry

We labeled *Chlorella vulgaris* cells with BODIPY 505/515 (4,4-Difluoro-1,3,5,7-Tetramethyl-4-Bora-3a,4a-Diaza-s-Indacene, Life Technologies, Grand Island, NY, USA) and measured their fluorescence with flow cytometry using a protocol adapted from Cirulis et al. [23]. Samples were prepared by first removing approximately 8 mL from the experimental culture without shaking or stirring. This ensured measurement consistency with the NMR measurement by characterizing only cells fully suspended within the medium as opposed to cells settled on the bottom of the culture vessel, as the suspended cells would be more likely to remain suspended in the reference region of the NMR sample tube during measurement.

Flow cytometry samples were then prepared by combining 930 *μ*L sodium phosphate buffer (40 mM, pH = 5.16), 10 *μ*L cell culture, 1 *μ*L BODIPY 505/515 stock solution (1 mg/mL in HPLC-grade dimethyl sulfoxide, stored frozen in 10-*μ*L aliquots), and 50 *μ*L CountBrite Absolute counting bead solution (Life Technologies). The BODIPY stock solution and counting bead solution were added in a darkened room to minimize photobleaching. We measured cellular fluorescence and scatter using an LSR II flow cytometer (BD Biosciences, San Jose, CA, USA) equipped with 488-nm and 355-nm lasers. For each flow cytometry event, we measured forward scatter (FSC) and fluorescence through the following fluorescence filters: FITC (530±15 nm excited at 488 nm), PE-Cy7 (780±30 nm excited at 488 nm), PerCP-Cy5.5 (695±20 nm excited at 488 nm), Pacific Blue (450±25 nm excited at 355 nm), and AmCyan (525±25 nm excited at 355 nm). Voltages for each detector are listed in the supporting information (S1 Table).

Cell events were gated according to their chlorophyll autofluorescence following the method developed by Cirulis et al. [23]. Briefly, chlorophyll autofluorescence was measured as infrared (PE-Cy7-A) and far red (PerCP-Cy5.5-A) fluorescence and plotted as cytograms of infrared vs. far red (sample cytograms in S2 fig). Events with insufficient infrared fluorescence (PE-Cy7-A < 200 FIU) were rejected as debris. The remaining cell events exhibited a consistent ratio of infrared vs. far red fluorescence attributable to chlorophyll, resulting in a narrow linear distribution of cell events on the flow cytogram. Other events such as counting beads and BODIPY dye precipitate exhibited a lower ratio of infrared vs. far red fluorescence, resulting in linear distributions further down on the infrared vs. far red cytogram that could be easily excluded by a diagonal boundary below the cell events. Additional gating details are described in the supporting information (S2 Fig caption). The fluorescence of cells labeled with BODIPY 505/515 was measured using the FITC filter. The voltage of the FITC detector was selected by characterizing unlabeled cells and adjusting the voltage so that between 5 and 100 out of 100,000 unlabeled cell events had FITC-A > 100 FIU. The initial cell concentration of each culture was calculated from the number of counting bead events according to the instructions from the manufacturer; detailed calculations and a description of this approach are included in the supporting information (S2 Table). At least 50,000 total events were measured for each flow cytometry sample, yielding at least 36,000 cell events.

### Liquid-state ^1^H NMR

Quantitative liquid-state ^1^H NMR spectra were measured at 600 MHz on a Varian INOVA 600 spectrometer (Varian, Inc., Palo Alto, CA, USA), controlled using VnmrJ 1.1C software, using the method of containing the reference standard in coaxial inserts developed by Henderson [39] and used by Davey et al. [4] to quantify TAGs *in situ* in *Chlorella* cultures. In addition, we implemented a multipoint calibration of the reference coaxial inserts instead of the single-point calibration used by Henderson [39] and Davey et al. [4]. NMR samples were prepared by first removing 12 mL of cell culture from the experimental culture without shaking or stirring the experimental culture. This ensured that only cells fully suspended within the medium were removed, as these suspended cells would be more likely to remain suspended in the reference region of the NMR sample tube during measurement. In order to increase cell concentration and minimize overlapping peaks from the tris(hydroxymethyl)aminomethane and acetate in the TAP medium, culture samples were centrifuged for 14 min at 3000 g and resuspended in 6 mL of an inorganic NMR analysis solution isotonic to the TAP medium and consisting of 15 mM NaCl, 700 *μ*M CaCl_2_, and 800 *μ*M MgSO_4_ · 7H_2_O in H_2_O.

Each final NMR sample was then prepared by pipetting 550 *μ*L of resuspended algae culture into an NMR sample tube (Wilmad 535-PP-7, Wilmad-LabGlass, Vineland, NJ, USA) and inserting a coaxial insert (Wilmad WGS-5BL, Wilmad-LabGlass) containing 100 *μ*L of a reference solution consisting of 2 mg/mL TMSP-d4 [3-(Trimethyl)propionic-2,2,3,3-d4 acid sodium salt, Sigma-Aldrich, St. Louis, MO, USA] and 60 *μ*g/mL Diethylenetriaminepentaacetic acid gadolinium(III) dihydrogen salt hydrate (Sigma-Aldrich) in D_2_O (99.9% D, Cambridge Isotope Laboratories, Tewksbury, MA, USA). Because the TMSP-d4 reference solution in the coaxial inserts occupies a different volume than the sample solution in the main NMR sample tube and is thus measured differently by the spectrometer receiver coils [39], it was necessary to determine the effective concentration of TMSP-d4 in the sample solution due to the inserts by characterizing the inserts in sample solutions with known composition. To do this, we calibrated each insert by measuring ^1^H NMR spectra for the insert placed in sample tubes containing 550 *μ*L of sucrose (≥ 99.5%, Sigma-Aldrich) in D_2_O for sucrose concentrations from 5 to 50 mg/mL. The area of the signal for the anomeric proton of sucrose (*δ* = 5.4 ppm) was normalized by the signal for the TMSP-d4 protons and correlated with the known sucrose concentrations—at least two concentrations for each insert, covering the range from 5 to 50 mg/mL—in order to calculate the effective TMSP-d4 proton concentration in the sample due to each insert. Sucrose calibration plots can be seen in the supporting information (S4 Fig).

NMR spectra for algae samples were measured after presaturation of the water peak using the PRESAT pulse sequence provided by Varian. To determine the optimal presaturation frequency for each sample, the presaturation frequency was initially placed at the location of the water peak in a standard ^1^H NMR spectrum and then varied to minimize the absolute value of the measured water peak in the PRESAT spectrum. Samples were characterized at 25°C with no spinning. Spectra were measured from *δ* = -3.0 to 14.0 ppm with a presaturation delay of 2.0 s, a presaturation power of 160 Hz, a 90° observer pulse width of 7.25 *μ*s, and a spectral width of 10191.1 Hz. Data sets of 16384 complex points were collected over an acquisition time of 1.608 s. To increase sensitivity, receiver gain was maximized for each sample with final values ranging from 46 to 54 dB. For final quantitative spectra, 1024 scans were averaged for each sample; however, acceptable spectra could also be acquired from as few as 64 scans.

We processed and analyzed the NMR spectra using the MestReNova software package (version 8.1.2, Mestrelab Research S.L., Santiago de Compostela, Spain). The time-domain free induction decay (FID) data was zero-filled to 32768 points and apodized with 0.5 Hz of exponential line broadening before Fourier transformation. Phase correction was adjusted manually for each spectrum. Baseline correction was achieved by fitting each spectrum to a 5^th^-order Bernstein polynomal. To determine the concentration of protons contained in algal TAG deposits, we integrated each spectrum over the TAG integral region from *δ* = 3.1 to 0.3 ppm and normalized the resulting intensity by the integral over the TMSP-d4 reference region from *δ* = 0.3 to -0.3 ppm. In order to remove the signal from peaks in the TAG integral region due to non-TAG components such as intracellular metabolites and residual acetate in the suspending medium (1.9 ppm), line fitting was applied to the TAG integral region. Peaks were fitted to generalized Lorentzian shapes with permissible linewidths from 0.25 Hz to 10.00 Hz. Fitted peaks with linewidths less than 3 Hz (0.005 ppm) were taken to be from non-TAG components, and the areas of these fitted peaks was subtracted from the overall integral over the TAG region. The normalized TAG integral was then multiplied by the effective TMSP-d4 proton concentration in the sample due to the coaxial insert in order to calculate the concentration of protons in the TAG integral region. The concentration of protons in the TAG integral region was also measured for the NMR analysis solution with no cells present, calculated to be 800±700 *μ*M, and subtracted from the concentrations of protons in the TAG integral region for each algae spectrum to determine the concentrations of protons per culture volume due to algal TAG deposits.

To convert the proton concentration to TAG concentration, we used the model TAG values developed by Davey et al. [4] based on chromatographic analysis of isolated algae neutral lipids. Their calculated model triacylglyceride contained an average of 85 protons per molecule in the TAG integral region and a molecular weight of 850 g/mol. We divided each measured TAG proton concentration by the model number of protons per molecule, multiplied the resulting lipid concentration (in mol/L) by the model molecular weight, and divided by the factor of concentration after resuspension in the NMR analysis solution, yielding TAG concentrations as mass per volume of culture. These values were then divided by the initial cell concentration in the culture as measured via flow cytometry using absolute counting beads in order to calculate the measured concentration of TAGs per cell. Detailed calculations are shown in the supporting information (S2 Table).

### Evaluation of measurement precision

We evaluated the precision of the flow cytometric measurements for mean BODIPY fluorescence and initial cell concentration by characterizing flow cytometric samples of replete and nitrogen-limited cells over a range of concentrations. Flow cytometry samples were prepared as described above, but with added cell culture volumes of 1, 5, 10, 25, and 50 *μ*L for each culture. We then characterized each sample via flow cytometry as described above and calculated the coefficient of variation of the measured BODIPY fluorescence and cell concentration for samples from each culture. We used the highest measured coefficient of variation for each measured parameter as its relative uncertainty.

We evaluated the precision of the ^1^H NMR measurements using the calibration for the reference coaxial inserts. As described above, each coaxial insert used for the ^1^H NMR measurements was calibrated by measuring NMR spectra for known concentrations of sucrose containing the insert. The absolute uncertainty of this NMR measurement in terms of proton concentration was taken as the standard error of the estimate for a linear regression, with no constant term, of sucrose concentration with respect to normalized intensity of the sucrose anomeric proton. Plots of measured flow cytometric parameters with respect to sample cell concentration (S3 Fig) and the sucrose calibration plots (S4 Fig) can be seen in the supporting information.

## Results and Discussion

**Fig 1:**
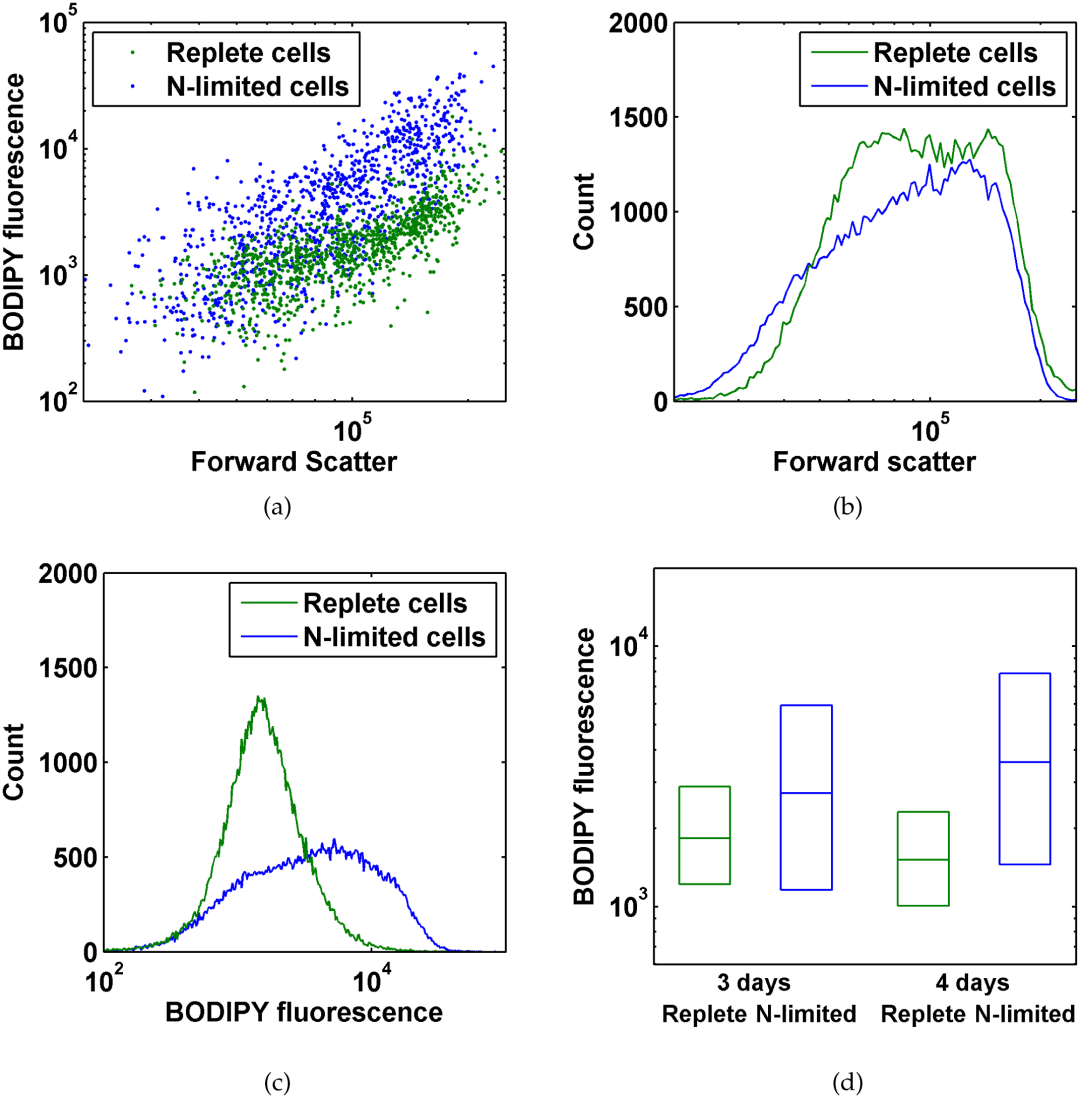
Flow cytometry measurements for *Chlorella vulgaris* cells labeled with BODIPY 505/515. All axes are logarithmic scale. (a) Flow cytogram of BODIPY fluorescence vs. forward scatter of cells after four days culturing in replete or nitrogen-limited medium. (b) Histogram of forward scatter for cells after four days culturing in replete or nitrogen-limited medium. (c) Histogram of BODIPY fluorescence for cells after four days culturing in replete or nitrogen-limited medium. (d) Box plot of medians and interquartile ranges (25th and 75th percentiles) for BODIPY fluorescence of cells after three and four days culturing in replete or nitrogen-limited medium.

Flow cytometry experiments revealed that *Chlorella vulgaris* cells cultured in nitrogen-limited medium to induce lipid accumulation [2, 17] exhibited greater BODIPY fluorescence per cell than cells cultured in nitrogen-replete medium (Fig 1). A cytogram of BODIPY fluorescence vs. forward scatter (Fig 1a) shows that cell events in each population with greater forward scatter (proportional to cell size) had greater BODIPY fluorescence, consistent with larger cells having higher absolute lipid content per cell. However, a histogram of forward scatter (Fig 1b) shows that the nitrogen-limited *C. vulgaris* cells exhibited approximately the same range of cell sizes as replete cells, whereas distributions of BODIPY fluorescence (Fig 1c and 1d) show that nitrogen-limited cells exhibited substantially higher lipid accumulation than replete cells.

**Fig 2:**
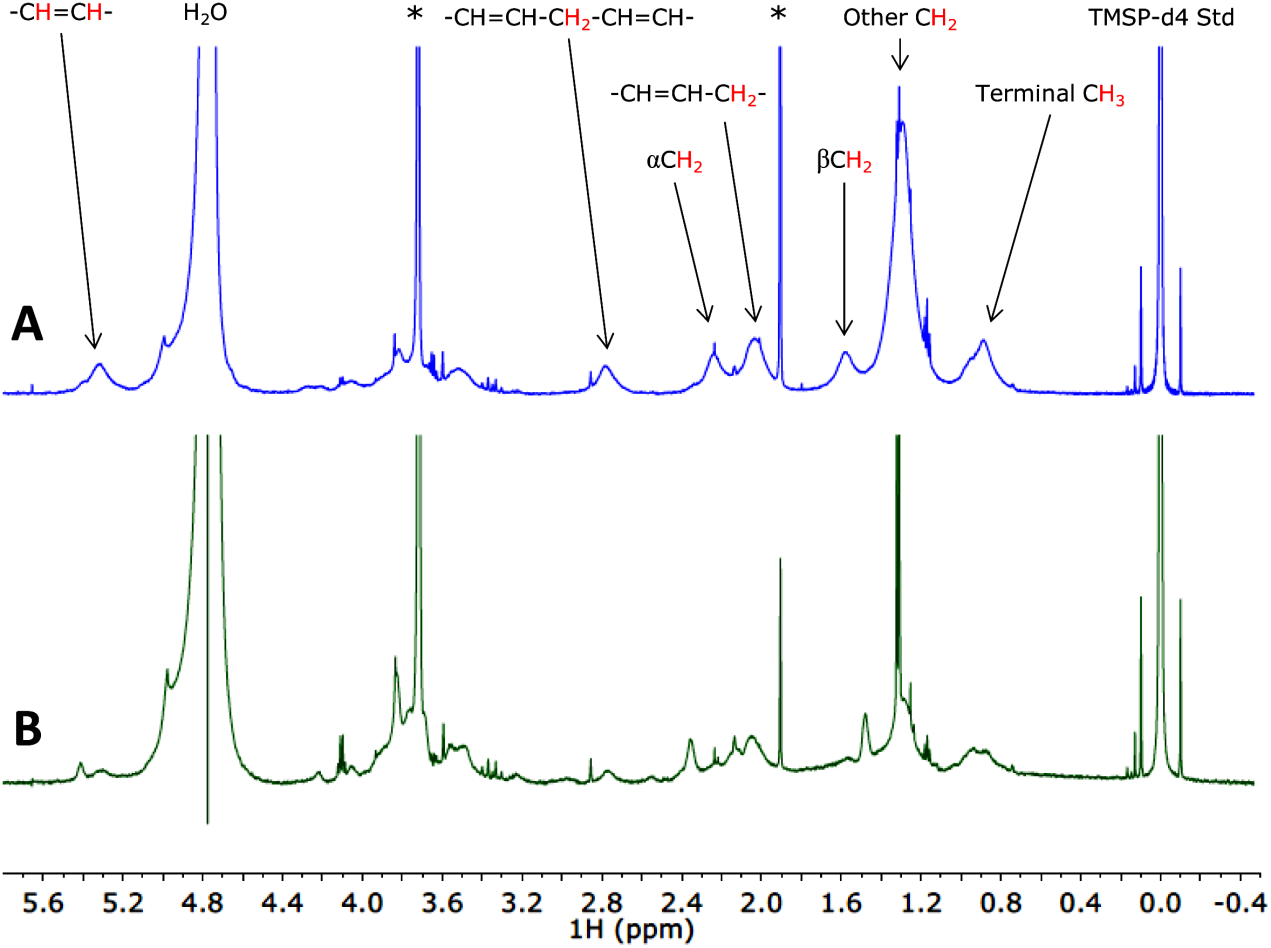
Representative measured NMR spectra for suspensions of intact *Chlorella vulgaris* cells cultured in (A) nitrogen-limited and (B) replete media. Samples resuspended in an inorganic NMR analysis solution, and characterized with a coaxial insert containing a TMSP-d4 reference as described in the Materials and Methods section. Peak assigments taken from Davey et al. [4]. Asterisks indicate peaks due to residual tris(hydroxymethyl)aminomethane (*δ* = 3.7 ppm) and acetate (*δ* = 1.9 ppm) from the TAP medium. For scale, note that the side peaks located at *δ* = ±0.1 ppm are ^13^C satellite peaks for the TMSP-d4 reference. The combined integral intensity of these peaks is 1.1% of total TMSP-d4 intensity, corresponding to approximately 120 *μ*M effective concentration.

**Fig 3:**
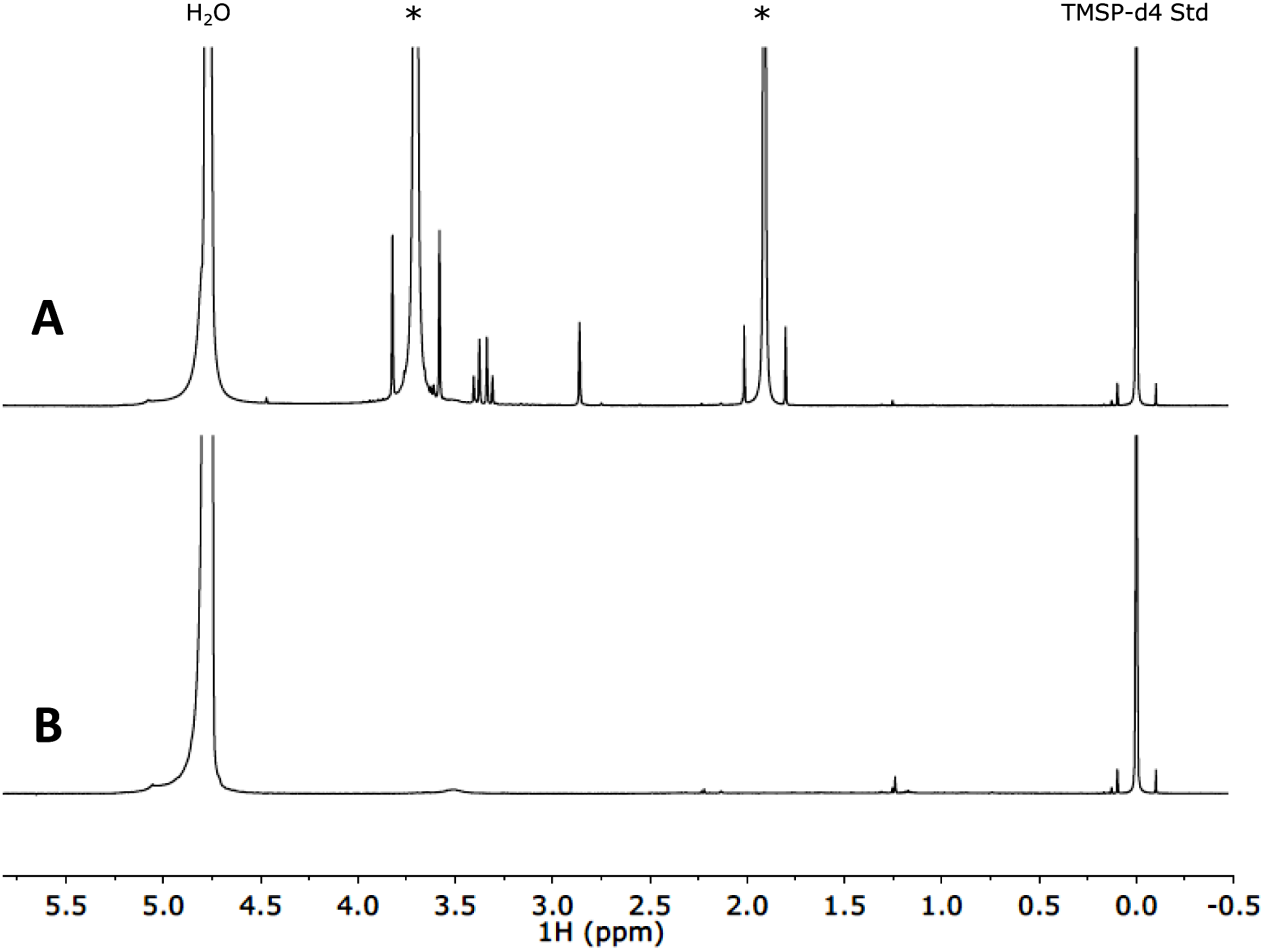
Background spectra for (A) the TAP medium that the algae were cultivated in and (B) the inorganic NMR analysis solution that the algae were resuspended in for ^1^H NMR measurements. Asterisks in the TAP medium spectrum indicate peaks from tris(hydroxymethyl)aminomethane (*δ* = 3.7 ppm) and acetate (*δ* = 1.9 ppm) that are also visible in our algae spectra due to residual tris(hydroxymethyl)aminomethane and acetate, and are similarly indicated in those spectra. As in Fig 2, the side peaks located at *δ* = ±0.1 ppm are ^13^C satellite peaks for the TMSP-d4 reference, with a combined integral intensity of approximately 120 *μ*M effective concentration.

In addition, NMR spectra of nitrogen-limited cells exhibited increased intensity in a series of peaks from *δ* = 3.1 to 0.3 ppm relative to spectra for unstressed cells cultured in replete media (Fig 2), indicating increased TAG deposits in the stressed nitrogen-limited cells. These TAG peaks are broader than those corresponding to the TMSP-d4 reference (*δ* = 0 ppm), intracellular metabolites (e.g. lactate doublet visible at 1.3 ppm), and residual growth medium components (*δ* = 1.9 and 3.7 ppm), as seen when comparing these spectra to the background spectra for TAP medium and inorganic NMR analysis solution shown in Fig 3. Moreover, the TAG peaks measured for cytoplasmic lipid deposits are also broader than those measured by Davey et al. [4] for isolated *Chlorella* neutral lipid fraction. The increased linewidth for peaks due to *in situ* TAG deposits is likely because the TAG molecules within algal cytoplasmic lipid deposits have decreased rotational mobility relative to isolated algal lipids; this increased microviscosity results in decreased transverse relaxation times T_2_ and broader spectral lines [42–45]. Additional line broadening may be due to differences in magnetic susceptibility between the cellular components and the growth medium, resulting in a heterogeneous applied magnetic field within the cells as has been suggested in previous liquid-state NMR characterization of intracellular lipid deposits by Davey et al. [4] and Millis et al. [46]. This increased linewidth for peaks due to TAG deposits facilitates easy removal of peaks due to non-TAG components via line fitting, as described in the Materials and Methods section.

By normalizing the integrated intensity of the TAG peaks by the reference peak due to the calibrated coaxial insert placed in each algae NMR sample, we calculated the TAG content per culture volume; this volumetric TAG content could then be converted to TAG content per cell using flow cytometric measurement of cell concentration for each culture (Table 1). We found that our calculated TAG content per cell was robust to processing of the time-domain NMR data, with little difference between values calculated from data after apodization, forward linear prediction, and no time-domain processing as shown in the supporting information (S3 Table).

Our NMR-derived values for TAG content (Table 1) are in agreement with previously measured values for *Chlorella* TAG content per cell and by culture volume. Vigeolas et al. [47] measured a TAG concentration of approximately 110 fg/cell for *Chlorella sorokiniana* cultivated mixotrophically in TAP medium up to mid-exponential phase, similar to our measured value for *C. vulgaris* 4 days after resuspension in fresh replete TAP medium. Our measured TAG concentrations for *C. vulgaris* during nitrogen limitation are within previously measured ranges of up to 1600 fg per cell for *C. vulgaris*[48] and up to 222 *μ*g per mL of culture volume for *Chlorella* sp. MAT-2008a [4]. We also detected TAG deposits in nitrogen-limited *Chlamydomonas reinhardtii* CC-125 cells but not replete *C. reinhardtii* cells (spectra in supporting information, S5 Fig), consistent with previous measurements indicating that wild-type *C. reinhardtii* contains very low TAG concentrations under unstressed conditions but accumulates TAGs during nitrogen limitation [49].

**Fig 4:**
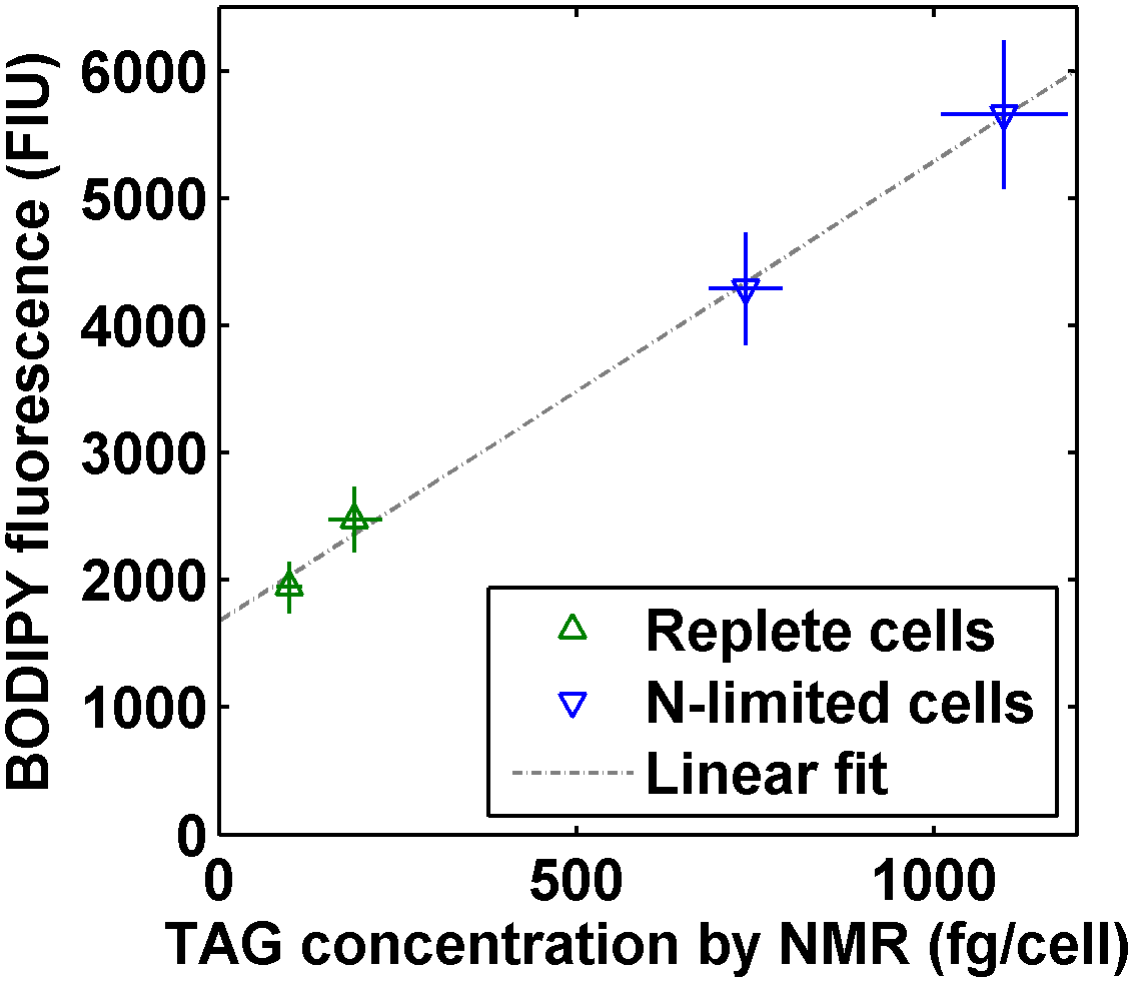
Mean BODIPY fluorescence per cell of each *Chlorella vulgaris* culture as measured via flow cytometry, plotted with respect to mean triacylglyceride (TAG) concentration per cell as measured via liquid-state ^1^H NMR. Error bars for TAG concentration by ^1^H NMR are calculated via propagation of uncertainty from the measured variation in estimated initial cell concentration and NMR quantification described in the evaluation of measurement precision section. Error bars for BODIPY fluorescence are taken directly from the measured variation in BODIPY fluorescence described in that section. Linear fit is ⟨BODIPY⟩ = 3.609[TAGs]+1682 (*r* ^2^ = 0.9974, *p* = 0.0013) for a linear regression of mean BODIPY fluorescence ⟨BODIPY⟩ in fluorescence intensity units (FIU) as a function of mean TAGs per cell by NMR [TAGs] in fg/cell, with 95% confidence intervals of [3.050, 4.168] for the slope and [1308, 2056] for the intercept.

When we compared the mean BODIPY fluorescence and corresponding average TAG content per cell from ^1^H NMR spectroscopy for each culture, there was an excellent correlation between the two measurements of lipid content as shown in Fig 4. The observed relationship and the Gaussian shape of the BODIPY histogram for the replete cells in Fig 1c are consistent with a linear fluorescent response of BODIPY in algal lipid deposits as observed previously [23]. A linear regression of mean BODIPY fluorescence 〈BODIPY〉 in fluorescence intensity units (FIU) as a function of TAGs per cell by NMR [TAGs] in fg/cell yielded the linear fit ⟨BODIPY⟩ = 3.609[TAGs] + 1682, with *r* ^2^ = 0.9974, *p* = 0.0013, and a standard error of the estimate of 106 FIU. This correlation compares favorably with the correlation measured by Cirulis et al. [23] (*r* ^2^ = 0.95) between BODIPY fluorescence measured by flow cytometry and fatty acid per cell measured via extraction and titration, and is comparable to correlations between Nile Red fluorescence and lipid content via extraction achieved by Chen et al. [22] using a plate reader assay (*r* ^2^ = 0.998) and Cirulis et al. [23] using flow cytometry (*r* ^2^ = 0.99). However, correlations between Nile Red and lipid content are subject to the aforementioned limitations of Nile Red such as non-specific fluorescence [27], spectral interference with chlorophyll autofluorescence [24, 28], and inconsistent uptake depending on cell wall structure [22], which complicate development of repeatable fluorescence protocols.

Based on our characterization of flow cytometry samples with cells from a given culture diluted to a range of sample cell concentrations, described in the section on evaluation of measurement precision, we determined that the flow cytometry protocol described here can be used for sample cell concentrations from 10^5^ to 10^7^ cells/mL with relative uncertainties of up to 10.3% for BODIPY fluorescence and 5.4% for estimated initial cell concentration. The standard error of the linear fit for BODIPY fluorescence as a function of TAGs by NMR was only 106 FIU, considerably less than the calculated uncertainties in BODIPY fluorescence for each culture shown in Table 1. This is expected because the relative uncertainty of 10.3% for BODIPY fluorescence was measured over a factor of 50 in sample cell concentration and the cell concentrations used in the main study varied by less than a factor of 3. Based on the calibration of the reference coaxial inserts used for the ^1^H NMR measurements, we estimated that the absolute uncertainties of the NMR measurements were 736 *μ*M and 452 *μ*M proton concentration for the inserts used, equivalent to 7.36 *μ*g and 4.52 *μ*g TAGs per mL of sample culture volume. As seen in Fig 4, the measured BODIPY fluorescence and TAG concentration by NMR were within their combined uncertainties from the linear fit for all cultures characterized.

The calculated linear fit indicates that there is additional BODIPY fluorescence (1682 FIU) which is not accounted for by the TAGs measured via liquid-state ^1^H NMR. We hypothesize that this is due to fluorescence from BODIPY molecules accumulated in non-TAG lipids such as phospholipids [28, 50]. Liquid-state NMR only samples protons in molecules that can rotate freely, such as TAGs present within cytoplasmic lipid deposits [4]. Protons in lipids with restrained motion, such as membrane phospholipids, are not detected because of line broadening due to chemical shift anisotropy and dipole-dipole interactions [42–46]. Our NMR measurements are in agreement with those of Davey et al. [4], who found that the lipid content of algae cultures measured via liquid-state NMR was less than that measured via fatty acid methyl ester derivation followed by gas chromatography (FAME-GC), which can quantitate both TAGs and ordered lipids such as membrane phospholipids. Use of semisolid NMR techniques such as high-resolution magic-angle spinning (HR-MAS) or intermolecular multiple-quantum coherence (iMQC) would facilitate quantitation of these ordered lipid molecules for improved calibration of BODIPY fluorescence [15, 46, 51]. However, the excellent correlation that we measure between BODIPY fluorescence and TAG content by liquid-state NMR suggests that the cellular concentration of these ordered lipids is unaffected by nitrogen limitation, with additional lipid accumulation mostly in the form of TAGs which can be quantitated via liquid-state NMR. This is in agreement with existing knowledge of the chemical composition of algal lipids synthesized in response to environmental stress [3]. Our current measurement approach facilitates develoment of an NMR-traceable flow cytometry protocol for quantification of cellular TAG deposits, with additional BODIPY fluorescence present which could be explained by the use of semisolid NMR techniques capable of quantifying ordered non-TAG lipids.

Our flow cytometry measurements suggest that nitrogen limitation induces lipid accumulation in only a subset of *C. vulgaris* cells. As we have shown that the mean BODIPY fluorescence of labeled cells in a culture measured via flow cytometry is strongly correlated with TAG content per cell by NMR, we can hypothesize that the distribution of measured BODIPY fluorescence for cells within a culture also indicates the distribution of lipid content for those cells. As seen in Fig 1c, *C. vulgaris* cells cultured in replete medium have a narrow distribution of BODIPY fluorescence, suggesting a relatively uniform lipid content. However, cells cultured in nitrogen-limited medium exhibit a wider range of BODIPY fluorescence that overlaps the range of fluorescences exhibited by replete cells. These measurements suggest that nitrogen-limited cells experience a range of lipid accumulation, with some cells accumulating no additional lipids. We observed this heterogeneity in lipid accumulation across cultures reuspended for 3 and 4 days, as seen in the box plot in Fig 1d.

Heterogeneous lipid accumulation in algae cells during nitrogen limitation was also observed by Davis et al. [5] in *Neochloris oleoabundans* cultures, and was even observed by Wang et al. [6] in isogenic *Nannochloropsis oceanica* cultures, indicating that the observed heterogeneity is probably not due to genetic differences within cultures. Lee et al. [52] used fluorescence microscopy to observe heterogeneous lipid accumulation in small populations (60 cells) of *C. vulgaris* cells immobilized in hydrogel microcapsules and labeled with BODIPY. Hitherto, a calibrated quantitative lipid measurement protocol and large sample populations (> 36,000 cells per sample) have not been used to demonstrate increased heterogeneity in the lipid content of *C. vulgaris* cells due to environmental stress. It remains unknown whether the observed heterogeneity is due to variations in light and nutrient conditions (e.g. carbon dioxide due to diffusion from the culture air-water interface) within the unstirred cultures or is an instance of transcriptional and phenotypic heterogeneity, which has been observed in other microbial organisms as a bet-hedging survival strategy and part of the overall stress response [53, 54].

## Conclusions

Single-cell fluorescence of *Chlorella vulgaris* cells labeled with BODIPY is strongly correlated with TAG content per cell as measured via liquid-state ^1^H NMR, with a coefficient of determination *r* ^2^ = 0.9974 that compares favorably with the correlation between a similar flow cytometry protocol and lipid content measured via extraction. In their current forms, the flow cytometry and NMR protocols described here could be used to develop an NMR-traceable flow cytometry protocol for quantification of cellular TAG deposits. We expect that semisolid NMR techniques such as high-resolution magic-angle spinning (HR-MAS) or intermolecular multiple-quantum coherence (iMQC) would facilitate detection of ordered non-TAG lipids in order to account for fluorescence from BODIPY accumulated in these non-TAG lipids. The increased variability in measured single-cell BODIPY fluorescence within each culture for nitrogen-limited cells suggests that only a subset of *C. vulgaris* cells accumulate additional lipids during nitrogen limitation. Heterogeneous lipid accumulation due to environmental stress has previously been observed in other algae species, but not *C. vulgaris*. Calibration of algal single-cell BODIPY fluorescence, measured via flow cytometry, using TAG content measured via liquid-state ^1^H NMR enables more accurate calibration of single-cell fluorescence to absolute lipid content, facilitating measurement of single-cell lipid content for development of improved algal strains and cultivation protocols for biodiesel production.

## Supporting Information

S1 Fig Growth curves for *Chlorella vulgaris* in TAP medium for the growth conditions used in this study. Growth curves plotted as (a) natural log of chlorophyll autofluorescence, excited at 440 nm and emission measured at 680 nm, and (b) absorbance at 640 nm. Chlorophyll autofluorescence was measured in order to calculate growth rate *μ* (in days^−1^) by linear regression of the equation for exponential growth, ln F = *μt*+ln F_0_, to measurements for natural log of fluorescence ln F during exponential growth, plotted with respect to time *t* measured in days; the regression constant ln F_0_ corresponds to the fitted value of ln F at *t* = 0. Absorbance was measured during stationary phase in order to determine the time of peak cell concentration. We measured fluorescence and absorbance using a Synergy H1 Hybrid Multi-Mode Microplate Reader (BioTek Instruments, Winooski, VT, USA). We measured the biomass at the end of stationary phase by removing 80 mL culture, concentrating it to 12 mL via centrifugation and resuspension, and lyophilizing the resuspended culture for five days in a FreeZone 4.5 Liter Benchtop Freeze Dry System (Labconco, Kansas City, MO, USA) before weighing the lyophilized biomass. From this measurement, we calculated a biomass concentration of 0.6 g/L at the end of stationary phase.
(TIF)

S1 Table Voltages used for flow cytometry detectors. (PDF)

S2 Fig Sample cytograms used for cell gating as described in the Materials and Methods section. Cell events are those events within the triangular gate in each PE-Cy7-A vs. PerCP-Cy5.5-A cytogram. Cell aggregates were excluded from these cell events by plotting cytograms of FSC-A vs. FSC-W for all cell events and rejecting events with FSC-W > 80. Counting bead events, shown on these cytograms as purple events, were gated as events with Pacific Blue-A > 2000 FIU and AmCyan-A > 200 FIU, as algae cells exhibited minimal fluorescence at these wavelengths.
(TIF)

S2 Table Calculations for initial cell concentrations and TAG concentrations. Initial cell concentration was calculated using the number of counting bead events. Each flow cytometry sample included 50 *μ*L of counting bead solution, containing approximately 54,000 counting beads. We could then use the number of counting beads and total sample volume (991 *μ*L) to calculate the volume of sample analyzed in each measurement, which, along with the number of cell events measured, yielded the sample cell concentration and initial concentration of the cell culture. Average TAG concentrations per cell according to NMR measurements were calculated using the approach described in the Materials and Methods section.
(PDF)

S3 Fig Measured variation in (a) BODIPY fluorescence and (b) estimated initial cell concentration for a range of sample cell concentrations. Sample cell concentration varied by adding different amounts of an individual cell culture to flow cytometry samples as described in the Materials and Methods section. If we consider sample cell concentrations between 10^5^ and 10^7^ cells/mL, then the coefficients of variation for BODIPY fluorescence are 7.9% for the replete culture and 10.3% for the nitrogen-limited culture, whereas the coefficients of variation for estimated initial cell concentration are 5.4% for the replete culture and 3.6% for the nitrogen-limited culture. We take the larger measured coefficient of variation for each parameter, 10.3% for BODIPY fluorescence and 5.4% for estimated initial cell concentration, as the relative uncertainty for that parameter when measured for sample cell concentrations from 10^5^ to 10^7^ cells/mL. If we include sample cell concentrations less than 10^5^ cells/mL then the coefficient of variation for the nitrogen-limited culture increases to 14.5% for BODIPY fluorescence and 8.6% for estimated initial cell concentration, so we do not claim that the flow cytometry protocol described here can be used for sample cell concentrations less than 10^5^ cells/mL.
(TIF)

S4 Fig Calibration curves for the TMSP-d4 reference coaxial inserts used for the ^1^H NMR measurements, denoted as (a) Insert A and (b) Insert B. As described in the Materials and Methods section, inserts were calibrated by measuring the peak intensity of the anomeric proton of sucrose (*δ* = 5.4 ppm) normalized by the TMSP-d4 reference peak for sucrose in D_2_O at concentrations from 5 to 50 mg/mL. We calculated the effective TMSP-d4 proton concentration for each insert as the slope of a linear regression with no constant term for sucrose concentration as a function of normalized intensity of the sucrose anomeric proton peak. For insert A the calculated effective TMSP-d4 proton concentration was 10.91 mM, with a standard error of the effective proton concentration of 49 *μ*M and a standard error of the estimate of 736 *μ*M. For insert B the calculated effective TMSP-d4 proton concentration was 11.09 mM, with a standard error of the effective proton concentration of 34 *μ*M and a standard error of the estimate of 452 *μ*M. We used the calculated effective TMSP-d4 proton concentrations to calculate the concentrations of TAG protons in algae samples containing these inserts, with absolute uncertainties equal to the standard error of the estimate from these calibration curves.
(TIF)

S3 Table Comparison of measured TAG contents for different processing of the NMR time-domain data: apodization, forward linear prediction, and no time-domain processing. (PDF)

S5 Fig Measured NMR spectra for suspensions of *Chlamydomonas reinhardtii* CC-125 cells characterized in their native growth media. Nitrogen-limited and replete cells were cultured as described in Bono et al. [17], with N-limited cells characterized 3 days after resuspension and replete cells characterized 1 day after resuspension. Cell-free medium is the supernatant for the N-limited cells. Spectra are an average of 512 scans. All other experimental details are as described for *C. vulgaris* samples.
(TIF)
