# Supplementary figures and images for: Measurement of Lipid Accumulation in Chlorella vulgaris via Flow Cytometry and Liquid-State ¹H NMR Spectroscopy for Development of an NMR-Traceable Flow Cytometry Protocol
Source: PLoS One. 2015 Aug 12;10(8):e0134846. doi: 10.1371/journal.pone.0134846 (PMC4534451; doi:10.1371/journal.pone.0134846)

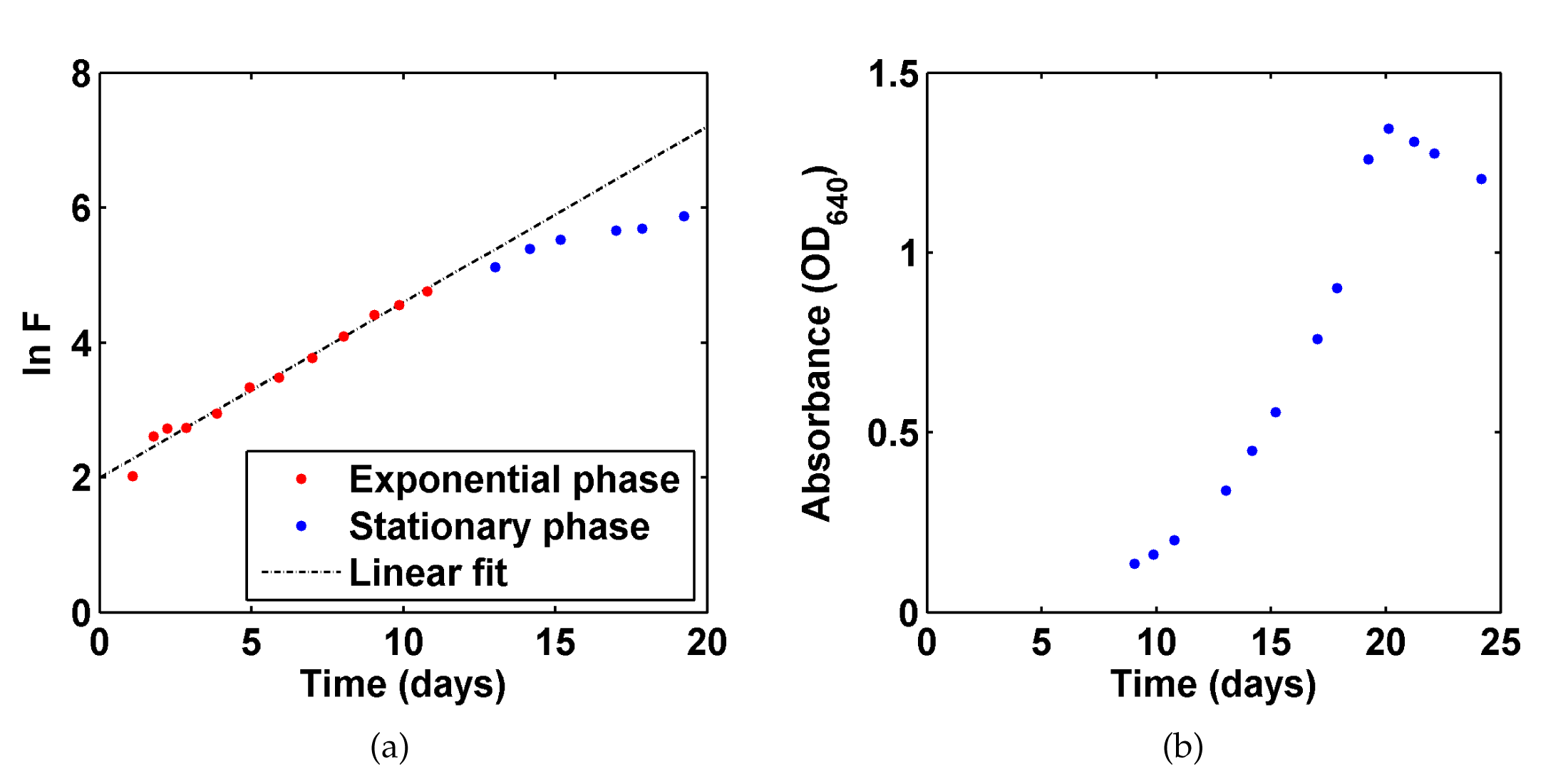

Supplement: S1 Fig — Growth curves plotted as (a) natural log of chlorophyll autofluorescence, excited at 440 nm and emission measured at 680 nm, and (b) absorbance at 640 nm. Chlorophyll autofluorescence was measured in order to calculate growth rate μ (in days−1) by linear regression of the equation for exponential growth, ln F = μt+ln F0, to measurements for natural log of fluorescence ln F during exponential growth, plotted with respect to time t measured in days; the regression constant ln F0 corresponds to the fitted value of ln F at t = 0. Absorbance was measured during stationary phase in order to determine the time of peak cell concentration. We measured fluorescence and absorbance using a Synergy H1 Hybrid Multi-Mode Microplate Reader (BioTek Instruments, Winooski, VT, USA). We measured the biomass at the end of stationary phase by removing 80 mL culture, concentrating it to 12 mL via centrifugation and resuspension, and lyophilizing the resuspended culture for five days in a FreeZone 4.5 Liter Benchtop Freeze Dry System (Labconco, Kansas City, MO, USA) before weighing the lyophilized biomass. From this measurement, we calculated a biomass concentration of 0.6 g/L at the end of stationary phase. (TIF) [file pone.0134846.s001.tif]

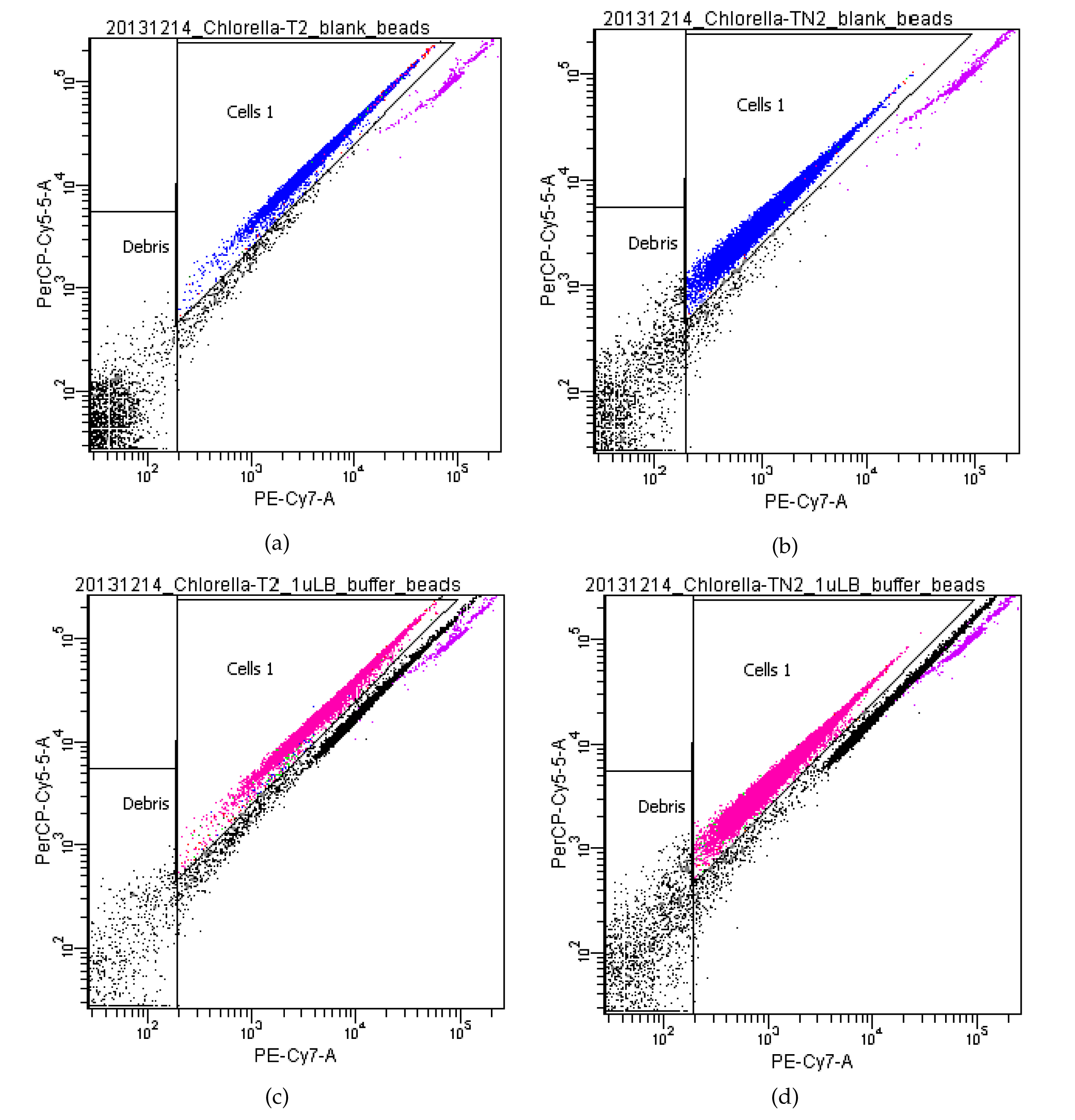

Supplement: S2 Fig — Cell events are those events within the triangular gate in each PE-Cy7-A vs. PerCP-Cy5.5-A cytogram. Cell aggregates were excluded from these cell events by plotting cytograms of FSC-A vs. FSC-W for all cell events and rejecting events with FSC-W > 80. Counting bead events, shown on these cytograms as purple events, were gated as events with Pacific Blue-A > 2000 FIU and AmCyan-A > 200 FIU, as algae cells exhibited minimal fluorescence at these wavelengths. (TIF) [file pone.0134846.s003.tif]

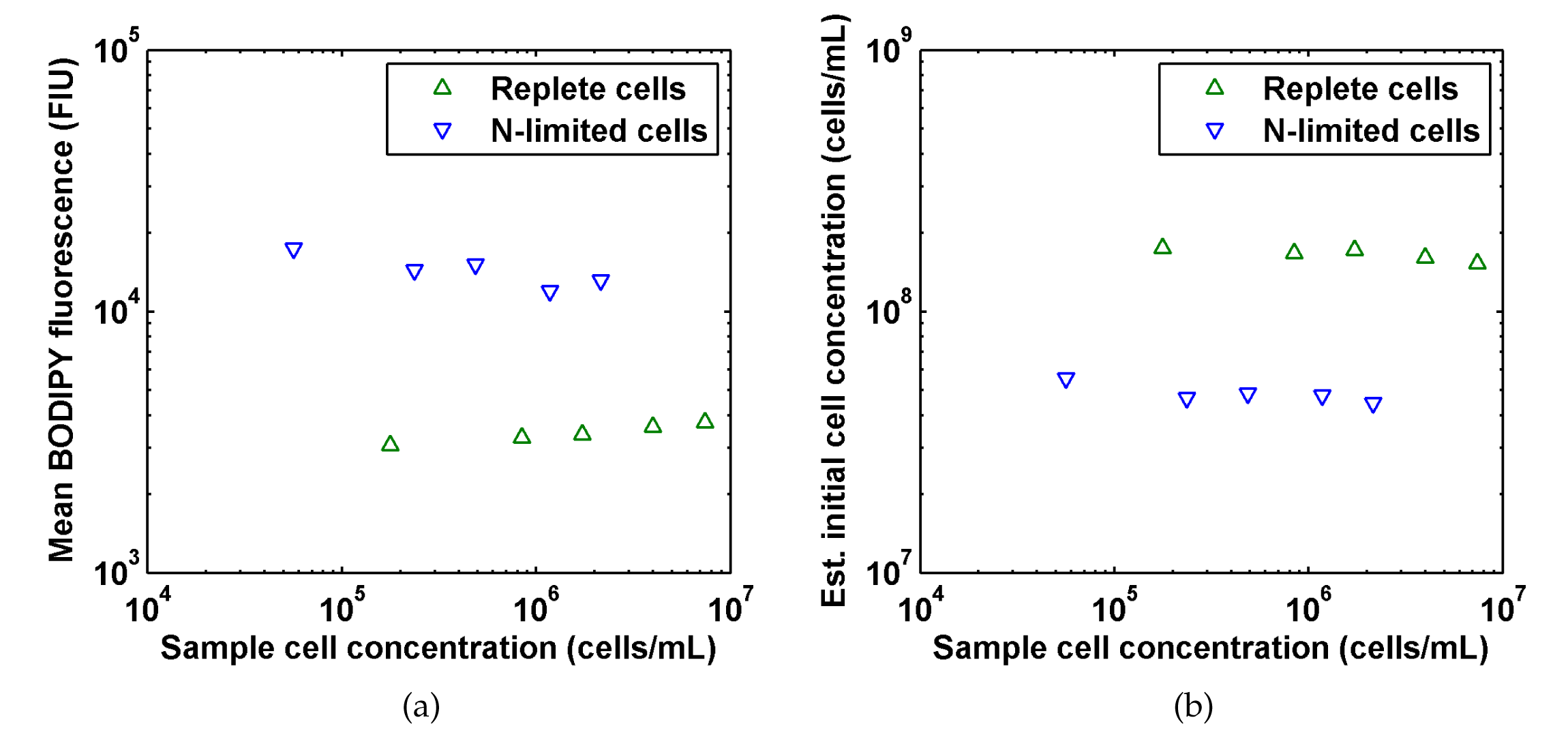

Supplement: S3 Fig — Sample cell concentration varied by adding different amounts of an individual cell culture to flow cytometry samples as described in the Materials and Methods section. If we consider sample cell concentrations between 105 and 107 cells/mL, then the coefficients of variation for BODIPY fluorescence are 7.9% for the replete culture and 10.3% for the nitrogen-limited culture, whereas the coefficients of variation for estimated initial cell concentration are 5.4% for the replete culture and 3.6% for the nitrogen-limited culture. We take the larger measured coefficient of variation for each parameter, 10.3% for BODIPY fluorescence and 5.4% for estimated initial cell concentration, as the relative uncertainty for that parameter when measured for sample cell concentrations from 105 to 107 cells/mL. If we include sample cell concentrations less than 105 cells/mL then the coefficient of variation for the nitrogen-limited culture increases to 14.5% for BODIPY fluorescence and 8.6% for estimated initial cell concentration, so we do not claim that the flow cytometry protocol described here can be used for sample cell concentrations less than 105 cells/mL. (TIF) [file pone.0134846.s005.tif]

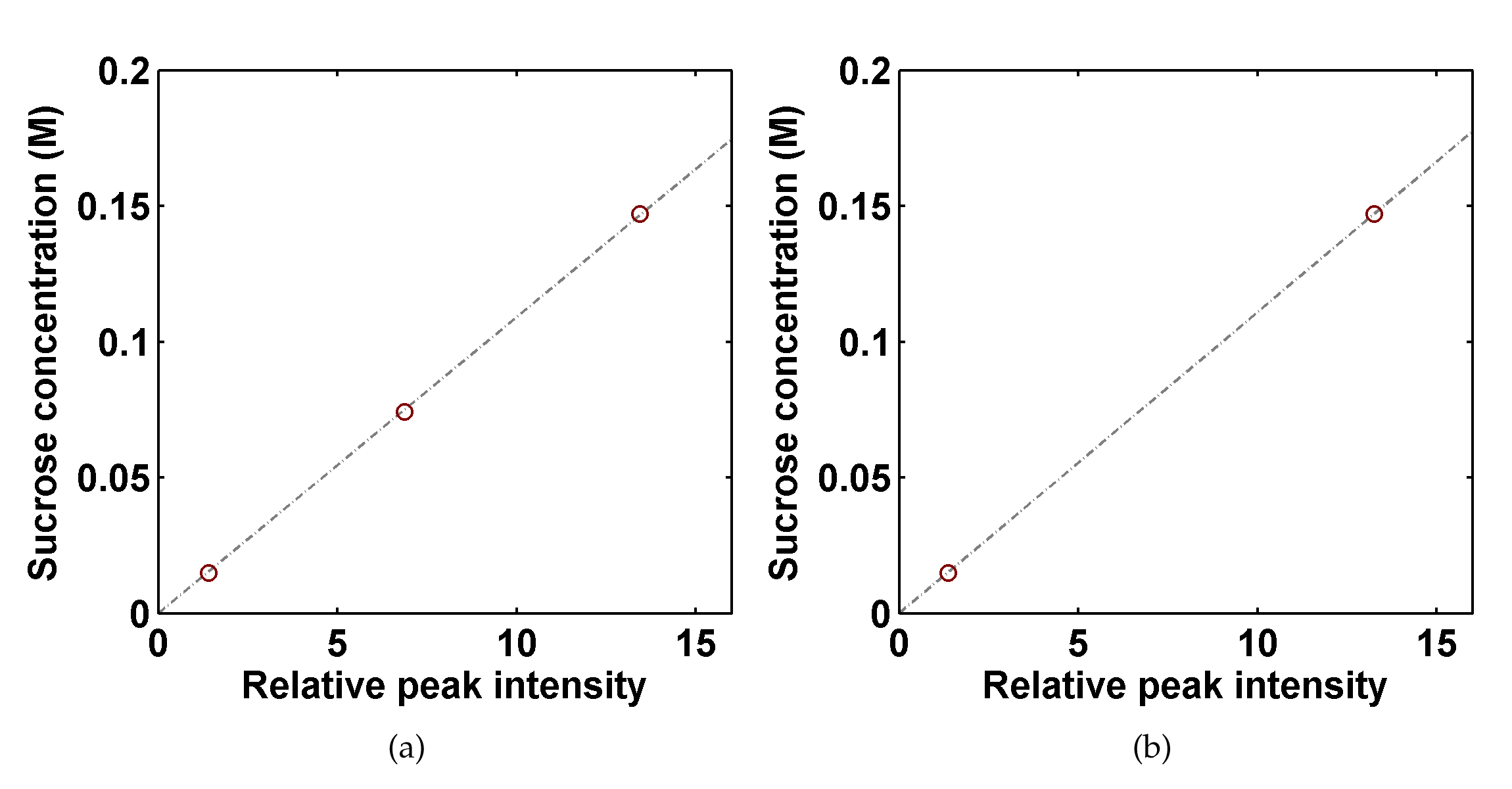

Supplement: S4 Fig — As described in the Materials and Methods section, inserts were calibrated by measuring the peak intensity of the anomeric proton of sucrose (δ = 5.4 ppm) normalized by the TMSP-d4 reference peak for sucrose in D2O at concentrations from 5 to 50 mg/mL. We calculated the effective TMSP-d4 proton concentration for each insert as the slope of a linear regression with no constant term for sucrose concentration as a function of normalized intensity of the sucrose anomeric proton peak. For insert A the calculated effective TMSP-d4 proton concentration was 10.91 mM, with a standard error of the effective proton concentration of 49 μM and a standard error of the estimate of 736 μM. For insert B the calculated effective TMSP-d4 proton concentration was 11.09 mM, with a standard error of the effective proton concentration of 34 μM and a standard error of the estimate of 452 μM. We used the calculated effective TMSP-d4 proton concentrations to calculate the concentrations of TAG protons in algae samples containing these inserts, with absolute uncertainties equal to the standard error of the estimate from these calibration curves. (TIF) [file pone.0134846.s006.tif]

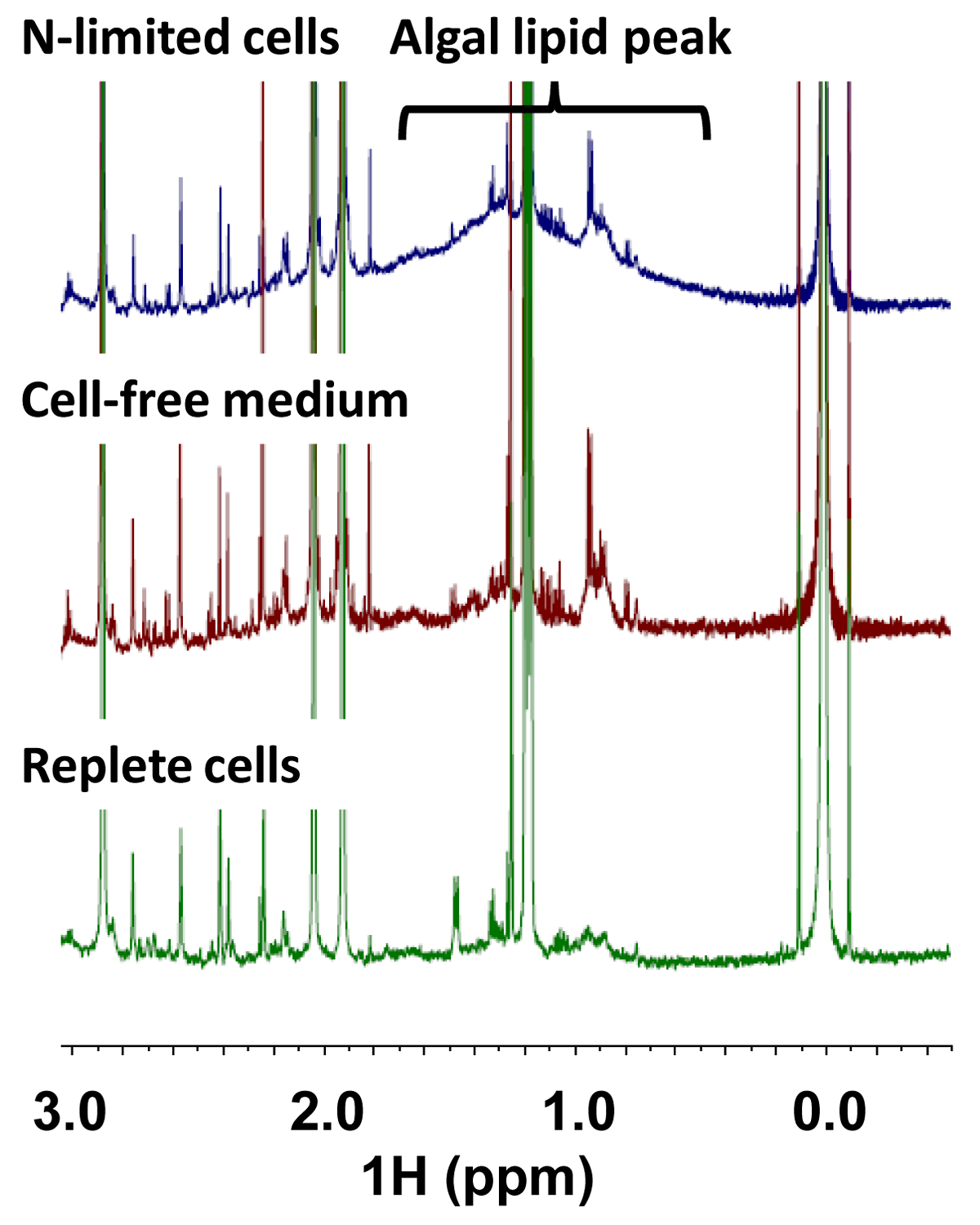

Supplement: S5 Fig — Nitrogen-limited and replete cells were cultured as described in Bono et al. [17], with N-limited cells characterized 3 days after resuspension and replete cells characterized 1 day after resuspension. Cell-free medium is the supernatant for the N-limited cells. Spectra are an average of 512 scans. All other experimental details are as described for C. vulgaris samples. (TIF) [file pone.0134846.s008.tif]
