# Supplementary material for: Measurement of Lipid Accumulation in Chlorella vulgaris via Flow Cytometry and Liquid-State ¹H NMR Spectroscopy for Development of an NMR-Traceable Flow Cytometry Protocol
Source: PLoS One. 2015 Aug 12;10(8):e0134846. doi: 10.1371/journal.pone.0134846 (PMC4534451; doi:10.1371/journal.pone.0134846)
Supplement: S1 Table — (PDF) [file pone.0134846.s002.pdf]

Voltages used for flow cytometry detectors.

Detector	Excitation (nm)	Emission (nm)	Voltage (V)	Use in study
Forward scatter (FSC)	-	-	451	Size measurement, singlet gating
FITC	488	530 ± 15	302	BODIPY fluorescence
PE-Cy7	488	780 ± 30	429	Infrared, cell gating x-axis
PerCP-C5.5	488	695 ± 20	429	Far red, cell gating y-axis
Pacific Blue	355	450 ± 25	302	Counting bead gating
AmCyan	355	525 ± 25	302	Counting bead gating
