# Supplementary material for: Measurement of Lipid Accumulation in Chlorella vulgaris via Flow Cytometry and Liquid-State ¹H NMR Spectroscopy for Development of an NMR-Traceable Flow Cytometry Protocol
Source: PLoS One. 2015 Aug 12;10(8):e0134846. doi: 10.1371/journal.pone.0134846 (PMC4534451; doi:10.1371/journal.pone.0134846)
Supplement: S2 Table — Initial cell concentration was calculated using the number of counting bead events. Each flow cytometry sample included 50 μL of counting bead solution, containing approximately 54,000 counting beads. We could then use the number of counting beads and total sample volume (991 μL) to calculate the volume of sample analyzed in each measurement, which, along with the number of cell events measured, yielded the sample cell concentration and initial concentration of the cell culture. Average TAG concentrations per cell according to NMR measurements were calculated using the approach described in the Materials and Methods section. (PDF) [file pone.0134846.s004.pdf]

Calculations for initial cell concentrations and TAG concentrations.

Days after resuspension	3	3	4	4
Culture medium	Replete	N-limited	Replete	N-limited
Cell events	41124	36419	83502	72457
Bead events	2142	2828	3285	6939
Sample volume characterized (μL)	39.3	51.9	60.3	127.3
Sample cell concentration (cells/μL)	1046	702	1385	569
**Initial cell concentration (10^6 cells/mL)**	104 ± 6	70 ± 4	137 ± 7	56 ± 3
NMR insert used	A	B	B	A
Effective TMSP-d4 proton concentration (mM)	10.91 ± 0.05	11.09 ± 0.03	11.09 ± 0.03	10.91 ± 0.05
Precision of NMR measurement (μM protons)	736	452	452	736
Normalized integral over lipid region	0.468	1.073	0.346	1.262
Area of narrow ($<$ 3 Hz) fitted lines in lipid region	0.044	0.089	0.042	0.067
Baseline correction	0.069	0.068	0.068	0.069
Corrected integral over lipid region	0.354	0.916	0.236	1.126
Sample TAG proton concentration (mM)	3.9 ± 0.7	10.2 ± 0.5	2.6 ± 0.5	12.3 ± 0.7
Sample TAG concentration (μg/mL sample)	39 ± 7	102 ± 5	26 ± 5	123 ± 7
Sample concentration factor	1.97	1.98	1.97	1.98
**Culture TAG concentration (μg/mL culture)**	20 ± 4	51 ± 2	13 ± 2	62 ± 4
**TAGs per cell (fg/cell)**	190 ± 40	740 ± 50	100 ± 20	1100 ± 90
