# Supplementary material for: Measurement of Lipid Accumulation in Chlorella vulgaris via Flow Cytometry and Liquid-State ¹H NMR Spectroscopy for Development of an NMR-Traceable Flow Cytometry Protocol
Source: PLoS One. 2015 Aug 12;10(8):e0134846. doi: 10.1371/journal.pone.0134846 (PMC4534451; doi:10.1371/journal.pone.0134846)
Supplement: S3 Table — (PDF) [file pone.0134846.s007.pdf]

Comparison of measured TAG contents for different processing of the NMR time-domain data before Fourier transformation. For apodization, we zero-filled the time-domain data to 32768 points and apodized with 0.5 Hz of exponential line broadening. For forward linear prediction, we used the Toeplitz method provided in MestReNova with 16358 basis points and 24 coefficients to predict an additional 16384 points in the time-domain data for 32768 points total. For no processing, the data was simply zero-filled to 32768 points with no apodization or forward linear prediction. Methods are compared in terms of the root mean square difference (RMSD) with respect to the results for apodization and the coefficient of determination r^2 for a linear regression with respect to the mean BODIPY fluorescence for each culture.

Time-domain processing method		Apodization	Forward linear prediction	No processing
TAGs per cell (fg/cell)	Replete, 3 days	189.2	180.9	174.9
	N-limited, 3 days	736.2	759.7	762.9
	Replete, 4 days	97.0	93.3	98.3
	N-limited, 4 days	1098.0	1099.9	1108.5
**TAGs per cell RMSD w.r.t. Apodization (fg/cell)**		–	12.6	16.1
**r^2 w.r.t. mean BODIPY fluorescence**		0.9974	0.9955	0.9945
